# Afterglow, TL and OSL properties of Mn^2+^-doped ZnGa_2_O_4_ phosphor

**DOI:** 10.1038/s41598-019-45869-7

**Published:** 2019-07-02

**Authors:** Andriy Luchechko, Yaroslav Zhydachevskyy, Sergii Ubizskii, Oleh Kravets, Anatoli I. Popov, Uldis Rogulis, Edgars Elsts, Enver Bulur, Andrzej Suchocki

**Affiliations:** 1 Department of Sensor and Semiconductor Electronics, Ivan Franko National University of Lviv, 107 Tarnavskogo St., Lviv, 79017 Ukraine; 2 Department of Semiconductor Electronics, Lviv Polytechnic National University, 12 Bandera St., Lviv, 79646 Ukraine; 3 Institute of Physics, Polish Academy of Sciences, Al. Lotnikow 32/46, Warsaw, 02-668 Poland; 4 Institute of Solid State Physics, University of Latvia, Riga, Latvia; 5 Physics Department, Middle East Technical University, 06800 Ankara, Turkey; 6 Institute of Physics, University of Bydgoszcz, Weyssenhoffa 11, Bydgoszcz, 85-072 Poland

**Keywords:** Materials science, Optical spectroscopy, Materials for devices

## Abstract

Zinc gallate (ZnGa_2_O_4_) spinel ceramics doped with Mn^2+^ ions was prepared by a solid-state reaction at 1200 °C in air. Manganese concentration was equal to 0.05 mol.% of MnO with respect to ZnO. Ceramics produced in this way show an efficient green emission at about 505 nm under UV or X-ray excitations, which is caused by Mn^2+^ ions. This green emission is observed also as a relatively long afterglow (visible to the naked eye in the dark for about one hour) after switching-off the X-ray excitation. Time profiles of the beginning of glow and afterglow have been studied together with thermally stimulated (TSL) and optically stimulated (OSL) luminescence. Experimental results demonstrate a presence of few types of shallow and deep traps responsible for the observed afterglow and TSL/OSL emission of the material. The possibility of pulsed optical stimulation and time-resolved OSL characteristics of ZnGa_2_O_4_: Mn^2+^ has been reported for the first time. The presented results suggest the ZnGa_2_O_4_: Mn^2+^ spinel as a promising material for further fundamental research and possibility of application as a green long-lasting phosphor or storage phosphor for TSL/OSL radiation dosimetry.

## Introduction

Oxide materials with spinel structure of general formula *AB*_2_O_4_ (*A* = Mg, Zn; *B* = Al, Ga) draw great attention due to their attractive optical-luminescent properties, high resistance to radiation damage as well as high thermal and chemical stability. All these properties make spinel compounds a suitable material for a wide range of applications, such as optical and insulating material in nuclear fusion reactors, vacuum fluorescent displays and electroluminescent displays, light emitting diodes (LEDs), solid-state lasers, UV photodetectors as well as storage and long persistent phosphors^1–17^.

A known feature of spinels is the presence of a high number of intrinsic point defects associated with the fact that in normal spinel structure only 1/8 of the tetrahedral sites are occupied by *A*^2+^ cations and 1/2 of the octahedral sites are occupied by *B*^3+^ cations. Such partial occupancy of cation sites, as well as a large amount of replacements (antisites) between *A* and *B* cations, causes numerous intrinsic defects that act as trapping centers for charge carriers created by radiation in the material^18^. The intrinsic cationic disorder depends strongly on the stoichiometry of the material and its preparation technology^18,19^ defining in such a way optical and luminescence properties of the material.

Phosphors activated with Mn^2+^ ions are of high importance among others spinel phosphors for various kinds of applications^20,21^. Mn^2+^ (3d^5^) ions, in particular in ZnGa_2_O_4_ spinel, produces a bright green color emission under the excitation by UV light or electron beam that can be applicable for vacuum fluorescent displays (VFDs), field emission displays (FEDs) and thin-film electroluminescent devices^22–25^.

Intrinsic trapping centers available in spinels can be exploited in order to receive a long persistent luminescence as well as the thermally (TSL) and optically (OSL) stimulated luminescence of the irradiated material^9–12^. In order to have an emission in the near-infrared range which is appropriate for biological applications, other transition metal activators like Ni^2+^ or Cr^3+^ can be used^6–8^.

Regarding the optically stimulated luminescence, it has become of high attention during the last decade as an alternative to TSL readout technique applicable for passive dosimetry of ionizing radiation^26–28^. The OSL technique for radiation dosimetry has a number of advantages over the TSL technique that leads to the gradual replacement of the conventional TSL dosimetry with OSL dosimetry that is observed nowadays. The specific requirements to OSL materials for dosimetry applications stimulate search and development of new materials applicable for this purpose. Among the perspective materials applicable for OSL dosimetry, various phosphors activated with Mn^2+^ ions are traditionally under study^12,29–39^.

Despite a considerable potential of spinels to exhibit optically stimulated luminescence as well as a long persistent luminescence, the materials with spinel structure activated with Mn^2+^ ions in this context remain scanty studied. TSL and time-resolved OSL studies of Mn^2+^-doped MgGa_2_O_4_ spinel ceramics have been done by us recently^12^. The ZnGa_2_O_4_ spinel that possesses much less inversion with respect to MgGa_2_O_4_ has not yet been studied from this point of view. Therefore this work’s aim is to study the afterglow, TSL and OSL properties of ZnGa_2_O_4_: Mn^2+^ in order to understand the ability to manage the properties of the material to fill better the requirements necessary for practical applications.

### Experimental details

Ceramic samples of zinc gallate doped with Mn^2+^ ions were obtained via a common high-temperature solid-state reaction technique. The zinc oxide (ZnO) and β-gallium oxide (β-Ga_2_O_3_) in the form of micropowders were used as initial materials. For doping with manganese, the MnO powder was used in the amount of 0.05 mol.% at the expense of ZnO. All reagents were at least 99.99% grade of purity. The initial powders with stoichiometric composition were grinded in an agate mortar for 6 hours. Obtained raw mixture was further pressed in а steel mold under the pressure of 150 kg/cm^2^. The annealing was performed at 1200 °C for 8 hours in the air. Thus samples in the form of tablets 4 mm in diameter and 1 mm thick were prepared. The X-ray diffraction measurements with the Rietveld refinement was reported previously^40^ demonstrate a pure ZnGa_2_O_4_ spinel phase of the ceramic samples prepared in such a way.

The photoluminescence (PL) and photoluminescence excitation (PLE) spectra were measured at room temperature using a Horiba/Jobin-Yvon Fluorolog-3 spectrofluorometer with a 450 W continuous xenon lamp for excitation and optical detection with a Hamamatsu R928P photomultiplier operating in the photon counting mode. The measured PLE spectra were corrected by the xenon lamp emission spectrum. The PL spectra were corrected for the spectral response of the spectrometer system used. All spectra were obtained with a spectral resolution of 0.5 nm.

A setup based on a SF-4A quartz monochromator was used to study the TSL curves. The microfocus X-ray tube with copper anode operated at 45 kV and 0.3 mA was used for irradiation of the samples. The temperature was monitored using a copper-constantan thermocouple clamped below the sample position. A linear heating with the 0.2 °C/s heating rate was provided with a RE-205 microprocessor temperature controller. The samples were investigated in vacuum cryostat to ensure the heating regime and to avoid an influence of environment.

The time-resolved OSL (TR-OSL) technique was used to study the optically stimulated luminescence properties of the material under pulsed stimulation. The measurement system used is described in details in refs. ^30,41^. For optical stimulation, a green LED (λ_max_ = 525 nm, power density at the sample position 10 mW/cm^2^) was used. An electromechanical shutter in front of the PMT was used because of the overlapping of the emission and stimulation wavelengths. The shutter was kept closed during the stimulation pulse. The TR-OSL signal was recorded using 0.655 s intervals upon starting of the stimulation pulse of 100 ms width. The TR-OSL emission was registered using a bialkali photocathode Hamamatsu R268P PMT in the photon counting mode with a blue-green bandpass filter (490 nm, bandwidth 10 nm) in front. Irradiation of samples was done with a Sr^90^/Y^90^ beta source at a dose rate at about 27 mGy/s.

## Results and Discussion

**Figure 1:**
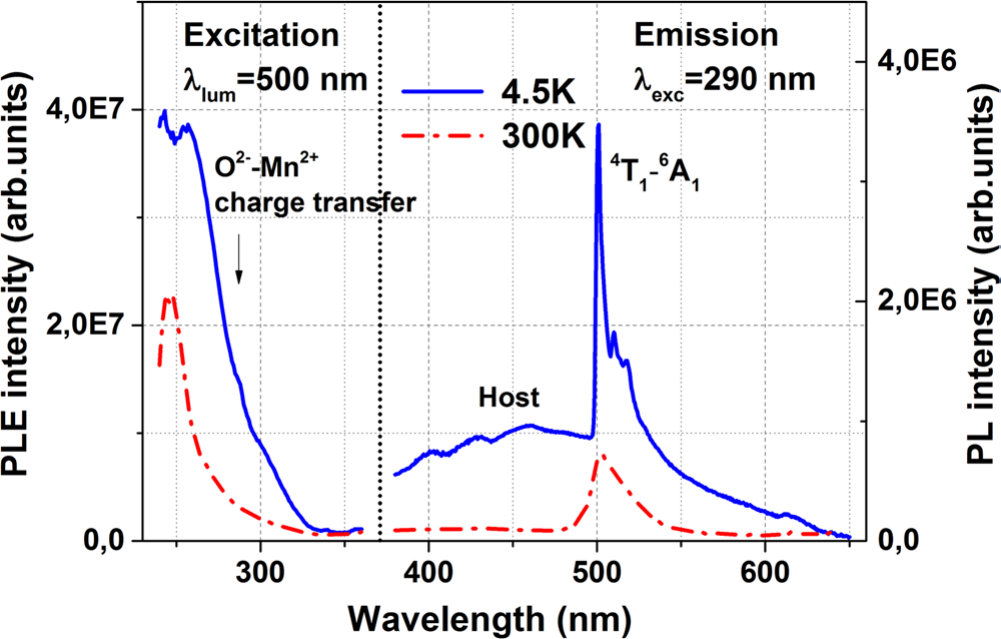
Photoluminescence excitation and photoluminescence spectra of ZnGa_2_O_4_: Mn^2+^ ceramics measured at temperatures 4.5 and 300 K.

The characteristic photoluminescence and photoluminescence excitation spectra of the studied ZnGa_2_O_4_: Mn^2+^ ceramic samples measured with high spectral resolution at low and room temperatures are shown in Fig. 1. The excitation of Mn^2+^–doped ZnGa_2_O_4_ ceramics monitored at 502 nm exhibits relatively strong excitation band in deep UV spectral range with a maximum at about 245 nm at room temperature which is related to the fundamental absorption of zinc gallate host. This indicates the recombination mechanism of Mn^2+^ ions excitation in the spinel host^18,42^. Slight shoulder in the 275–320 nm range is assigned to a charge transfer from oxygen to manganese ions^23–25,40^. Cooling down to 4.5 K leads to the increase in the efficiency of excitation as well as to the redistribution of intensity in favor of the O^2−^ → Mn^2+^charge transfer band. It should be noted that some weak excitation in the 360–480 nm wavelength range related to the spin-forbidden *d-d* electronic transitions in Mn^2+^ ions was also observed at 4.5 and 300 K. But its intensity is more than an order of magnitude lower than the excitation at 240–245 nm.

As it is seen from Fig. 1, the excitation of ZnGa_2_O_4_: Mn^2+^ ceramics with 290 nm that is below the energy band gap of the host and hits into the charge transfer band produces only the activator emission at 480–550 nm at room temperature. The same but much weaker emission is observed at excitation in relatively narrow peak 410 nm assigned to *d-d* electronic transitions in Mn^2+^ ions in tetrahedral sites of the lattice. Here should be noted, that Mn^2+^ emission band has an asymmetric line shape which indicates on the complex nature of this band. The significant changes in the emission spectra are observed at lower temperatures. At 4.5 K, two types of emissions can be clearly distinguished. Besides the emission of Mn^2+^ ions at 490–550 nm, the complex intense emission band at 380–490 nm related to host defects of the spinel structure is also observed. Moreover, the shape of the spectrum of the activator was also significantly changed at low temperature. In particular, the green luminescence of Mn^2+^ ions has been increased accompanied by better resolved fine structures. The UV-blue host emission is emitted from the self-activation center of octahedral Ga–O group in the spinel lattices^24^. S.S. Yi *et al*. also showed that blue emission of ZnGa_2_O_4_ is related to Ga–O group^43^. Authors of this work refined intra-shell electronic transitions of Ga^3+^ cations in the PL spectra. This explains the temperature behavior of the UV-blue emission band in the case of presented studies. The green emission at 500–550 nm is typical for the divalent manganese ions in the regular fourfold tetrahedral coordination (*A* site) and corresponds to the ^4^T_1_ → ^6^A_1_ transition in Mn^2+^ ion^22–25^. We assume that the 502 nm emission line is due to Mn^2+^ ions in the high symmetry tetrahedral sites. At the same time, the satellite emission lines at 510 and 517 nm observed in the studied ZnGa_2_O_4_: Mn^2+^ ceramics at the low temperature we attribute to Mn^2+^ ions in sites with lower symmetry, for example, ones distorted by oxygen vacancies.

Here it should be noted that 245 nm excitation wavelength is deep in the “band-to-band” region taking into account band gap at the level of 4.3 eV^44^. Therefore, the PL spectrum at this excitation shows the intensity of the green emission at about 50% less at room temperature than when excited in the charge transfer band. Moreover, it is also similar to the X-ray luminescence spectrum, which indicates the recombination mechanisms of excitation in Mn^2+^ ions as mentioned above.

**Figure 2:**
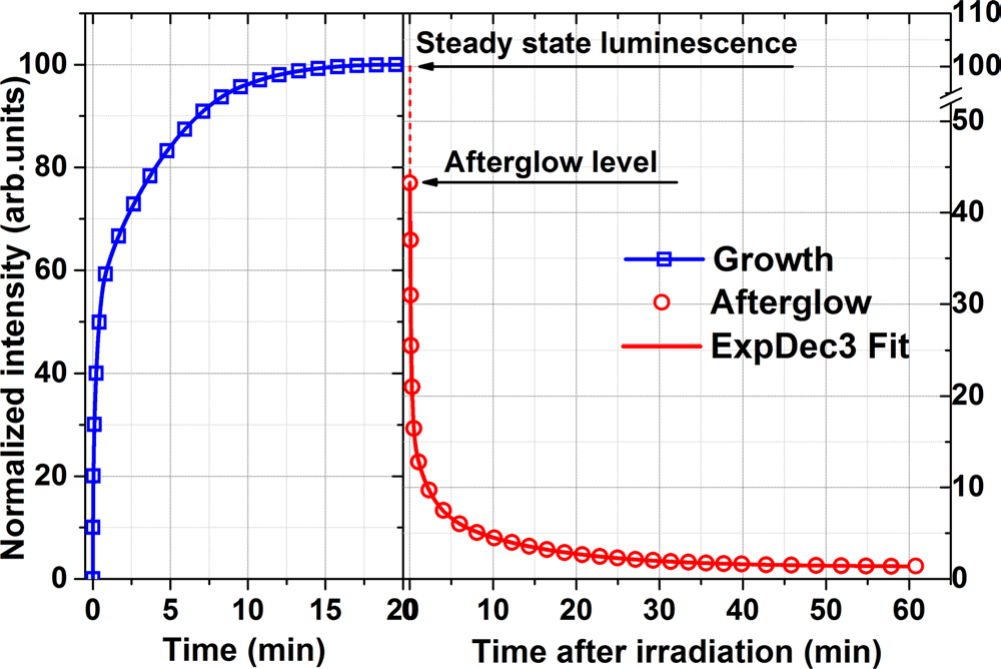
The beginning of glow (left) and afterglow (right) curves of the studied ZnGa_2_O_4_: Mn^2+^ ceramics monitored at 505 nm under (after) X-ray exposure at room temperature.

Besides the intense photoluminescence and X-ray luminescence, ZnGa_2_O_4_: Mn^2+^ phosphor exhibits green long persistent phosphorescence after the removal of the excitation source. The X-ray luminescence intensity growth and afterglow decay curve obtained in the continuous registration mode at 505 nm monitoring are shown in Fig. 2. At the beginning (just after the switching-on of the X-ray excitation), the observed emission increases extremely fast, after that, the growth becomes slower and the maximal emission intensity is reached after about 15 minutes of X-ray exposure. Further exposure doesn’t change the steady-state level of the emission intensity. Accordingly, 15 min of X-ray exposure is enough to reach the level of steady-state X-ray luminescence and 5 more minutes was taken to fill all trapping states.

**Figure 3:**
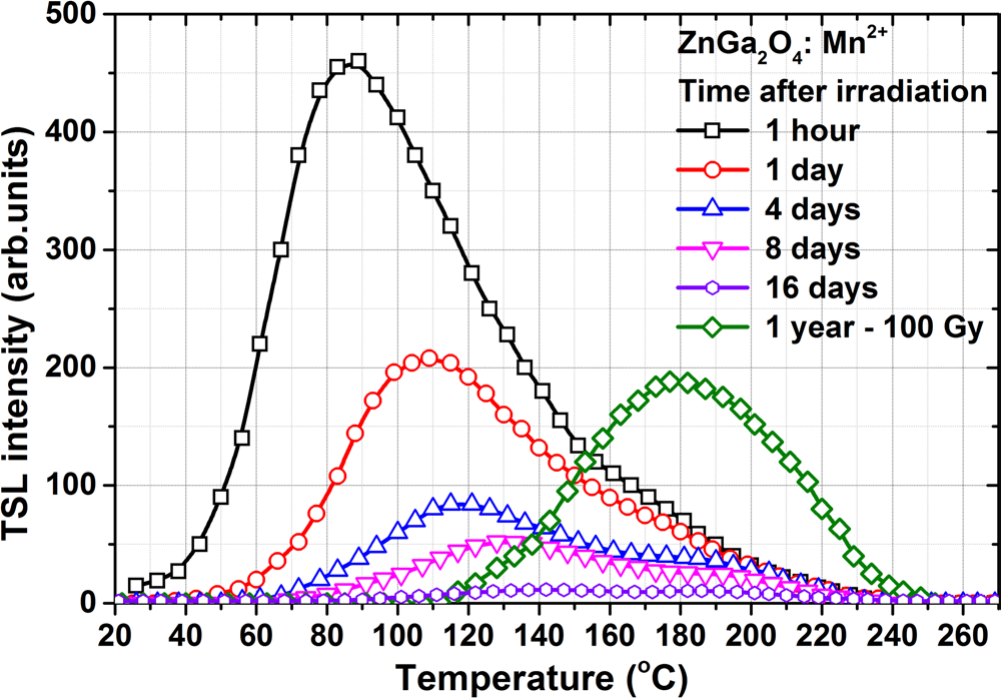
TSL glow curves of ZnGa_2_O_4_: Mn^2+^ ceramics registered at 505 nm for various time periods after the X-ray irradiation at room temperature. The heating rate is 0.2 °C/s. Green curve − after one-year storage following a 100Gy irradiation with the Sr90/Y90 beta source.

ZnGa_2_O_4_: Mn^2+^ ceramics after the X-ray exposure for 20 min demonstrates a bright and relatively long afterglow of green color which is visible to the naked eye in the dark for one hour. After the X-ray exposure switched off, the emission intensity drops to the initial afterglow level which is approximately 44% of the steady-state intensity of luminescence. The afterglow curve demonstrates a rapid decay at the beginning and then the glow lasting for a longer time. In particular, about 3% of the steady-state emission intensity is still registered after 60 minutes of observation. The same 3% of the afterglow intensity corresponds to the first point of the first TSL glow curve presented in Fig. 3. The observed afterglow decay curve was fitted by the triple-exponential decay:

1 $$I(t)={I}_{1}{e}^{t/{\tau }_{1}}+{I}_{2}{e}^{t/{\tau }_{2}}+{I}_{3}{e}^{t/{\tau }_{3}}$$

there *I(t)* is the luminescence intensity at the decay time *t*; *I*_1_*, I*_2_*, I*_3_ are the initial intensities (at *t* = 0) of each component; *τ*_1_*, τ*_2_ and *τ*_3_ are the time constants that describe the rate of certain decay. The fitting parameters are given in the Table 1. The observed afterglow can be explained by the charge carrier release from shallow traps at room temperature which is enough to move electrons or holes from a trap level in bandgap to an appropriate band, i.e. conduction or valence band, respectively. Anyhow, the triple-component decay clearly points on a complex mechanism and few types of shallow traps involved in the afterglow process.

**Table 1:** Triple-exponential fitting of the afterglow curve of the ZnGa_2_O_4_: Mn^2+^ ceramics. Overall coverage (Adj. R-Square) 0.99822.

	I_1_, arb. u.	τ_1_, min	I_2_, arb. u.	τ_2_, min	I_3_, arb. u.	τ_3_, min
Value	27.436	0.208	10.748	3.738	4.500	40.793
Standard Error	1.80e–01	1.42e–03	4.82e–02	3.68e–02	3.81e–02	4.54e–01

Thermally stimulated luminescence (or just TSL) glow curves of the ZnGa_2_O_4_: Mn^2+^ ceramics recorded after various periods of time after the X-ray exposure are shown in Fig. 3. These measurements were carried out to define the room-temperature stability of the TSL glow and to estimate the distribution of the trapping centers. During this experiment, the samples all the time were kept in the dark. All the TSL measurements (except the last one after 1-year storage with a higher dose) were carried out in identical conditions as the sample was left in the cryostat for whole period of measurements and wasn’t taken out. Here the X-ray irradiating during 20 min was used that corresponds to the absorbed dose of about 7Gy.

As it is seen from Fig. 3, the TSL glow of ZnGa_2_O_4_: Mn^2+^ is rather complex and consists of a few overlapping peaks. The first TSL curve was measured 1 hour after irradiation to avoid afterglow influence on the shape of the curve. This curve has a maximum at about 85 °C and the shoulder on the high-temperature side. After one day storage, the TSL glow reveals as a peak having maximum at about 110 °C at the heating rate used. The shift of the TSL maximum towards higher temperatures as the storage time increases indicates that a distribution of traps of different depths contributes to the TSL. Because of low-temperature TSL peak strongly disappears after storage of samples for one day it can be assumed that shallow traps responsible for this peak have very short lifetime and are emptied for a really short period of time. Simultaneously the high-temperature peaks decrease slowly, especially when changing storage periods from 4 to 8 days. The observed TSL glow is suppressed almost completely after 16 days of storage. This differs the material under study from MgGa_2_O_4_: Mn^2+^, for which a relatively large amount of TSL still remains for a peak at about 150 °C after 16 days of storage^12^.

The complex nature of TSL glow at X-ray exposure was confirmed for spinel oxide compounds in^4,11,12,23,24^. In particular, the shift of TSL peak for various MgAl_2_O_4_ samples to higher temperatures in the range 20–220 °C using pre-heating to suitable temperatures was shown in^11^. However, TSL features of MgGa_2_O_4_: Mn^2+^ compound shows a more complex shape of the TL curve^12^. It is related first of all that MgGa_2_O_4_ compound possesses in a huge amount of antisite defects (when Mg and Ga atoms are exchanged by positions) while the zinc gallate has completely normal spinel structure and free of this type of defects^12,40^.

Earlier, Uheda et. al.^23^, reported persistent luminescence of Mn^2+^ doped in ZnGa_2_O_4_. These authors have found that annealing at high temperatures leads to intense evaporation of ZnO with the formation of a high concentration of zinc vacancies (V_Zn_^2−^). TSL glow curve of ZnGa_2_O_4_: Mn^2+^ investigated by authors^23^ showed an intense peak at about 320 K that corresponds to release of holes from the V_Zn_ - defects. This suggestion is also supported with results of ZnGa_2_O_4_: Cr^3+^ TSL glow investigation obtained by^45^. At the same time, evaporation of ZnO during the annealing also leads to the generation of oxygen vacancies^46^. Accordingly, the high-temperature peaks are most probably related to electron release from oxygen vacancies defects which can capture two electrons. Capturing a single electron, the oxygen vacancy creates the F^+^- center, which is commonly observed in oxide materials. Further capture of the electrons from the conduction band modifies the state of the oxygen vacancy (F-center). Thus, taking into account the TSL glow curves and earlier reported results by^23^, it is possible to suggest that afterglow origins from a shallow trap created by zinc vacancies.

The TSL glow curve of ZnGa_2_O_4_: Mn^2+^ after one-year storage following a 100Gy irradiation with the Sr^90^/Y^90^ beta source is also shown in Fig. 3 for comparison. It should be noted that the TSL intensity is about half of that one has been in a 1 hour after X-ray exposure for 20 minutes. Moreover, the TSL peak at 180 °C observed here indicates that beside the relatively shallow traps responsible for the glow at temperatures slightly above the room temperature, the deep traps responsible for the 180 °C peak also exist in ZnGa_2_O_4_: Mn^2+^. This observation indicates a principle opportunity to use ZnGa_2_O_4_: Mn^2+^ phosphor as a dosimetric material. However, additional studies aimed to estimate a relative sensitivity and fading characteristics of the TSL peak at 180 °C are required.

**Figure 4:**
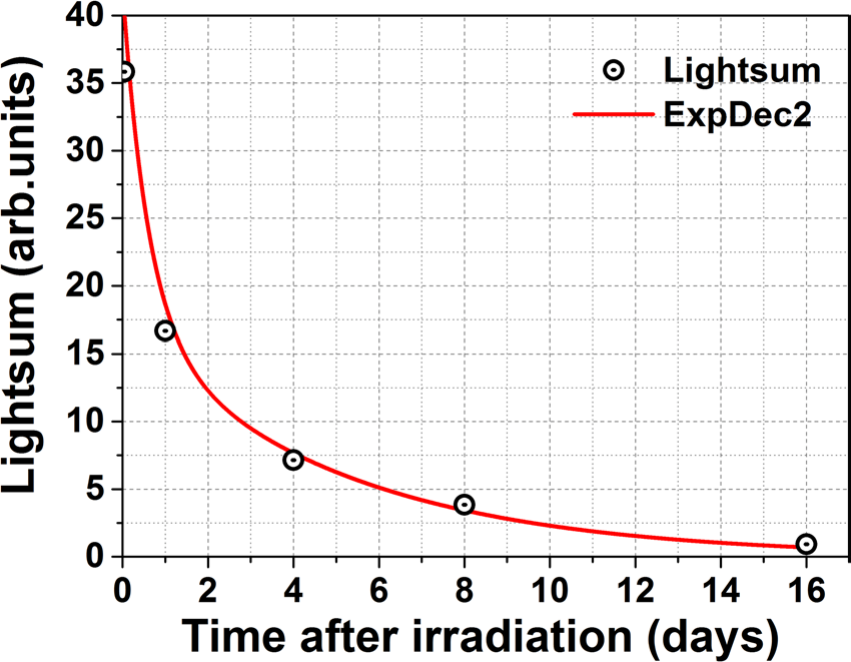
Thermal fading of the total lightsum during dark storage of ZnGa_2_O_4_: Mn^2+^ ceramics at room temperature.

Meanwhile, the fading of the total lightsum (determined as the area under the TSL glow curve) of the material under study versus storage time is shown in Fig. 4. The observed fading of TSL can be fitted by the bi-exponential decay (solid line in Fig. 4). As it is evident from the figure, more than 70% of the total lightsum fades already after 4 days of storage that corresponds to the fast exponential component of the fitting. The decay after 4 days seems to be more slow exhibiting a less relative decrease of the residual lightsum.

**Figure 5:**
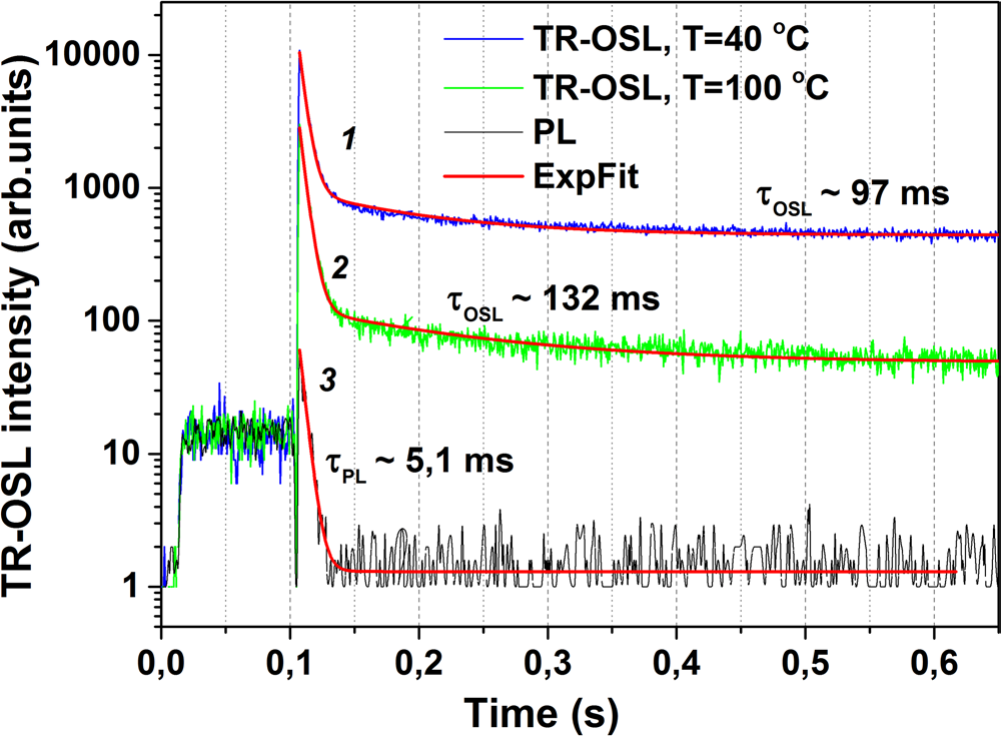
Typical TR-OSL decay curves obtained for ZnGa_2_O_4_: Mn^2+^ ceramics at room temperature after 10Gy β–irradiation and next preheating during 2 min at 40 °C (1) and 100 °C (2), as well as an analog of PL decay for the non-irradiated sample (3).

The TR-OSL decays obtained for ZnGa_2_O_4_: Mn^2+^ after 10Gy irradiation and preheating for 2 min at 40 and 100 °C are shown in Fig. 5 (as curves 1 and 2, respectively). Thereafter, the TR-OSL decay curves were recorded at room temperature. In order to reach a better signal-to-noise ratio, the signal was accumulated for 10 stimulation pulses. As seen from the figure, TR-OSL decay curves exhibit a fast initial decay followed by a slower one. An analysis made by curve fitting using a bi-exponential decay function gives the slow component’s lifetimes as 97 and 132 ms for 40 and 100 °C preheats, respectively. The fast decay component with the lifetime 5.1 ms corresponds to the Mn^2+^ photoluminescence decay. This is evident from the curve 3 in Fig. 5 for the non-irradiated sample (heated up to 650 °C and stored in darkness) measured in the same experimental conditions as curves 1 and 2. Thereby the PL signal measured herewith represents a background for TR-OSL signal. Similar PL lifetime of 4.2 ms for Mn^2+^ ions in ZnGa_2_O_4_: Mn^2+^ nanophosphor have been reported in^22^.

**Figure 6:**
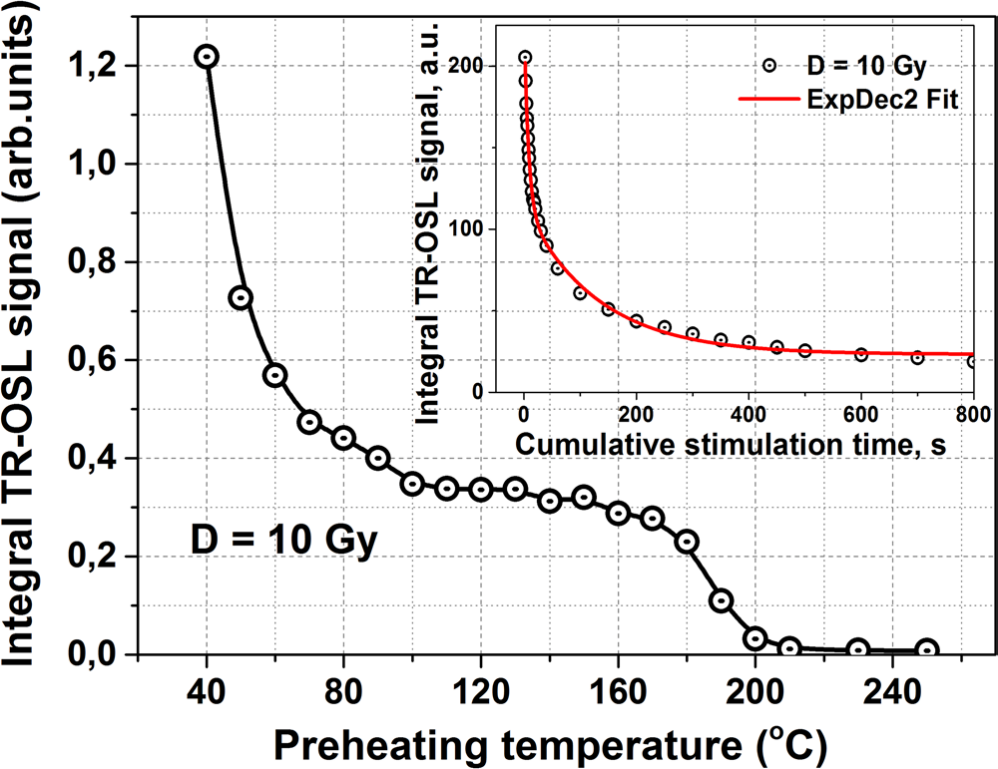
The integral TR-OSL signal of ZnGa_2_O_4_: Mn^2+^ ceramics after 10Gy irradiation depending on the preheating temperature. The inset represents the optical bleaching of the TR-OSL signal as a function of cumulative stimulation time (the solid line shows a fitting by double-exponential decay).

Temperature stability of the OSL signal intensity, which presents the integrated TR-OSL signal during the optical stimulation as a function of the preheating temperature, is shown in Fig. 6. The integrated OSL signal was taken as area under the measured TR-OSL curve in the 0.655 s time interval. The TR-OSL signal curve has been corrected on the contribution PL signal as a background in the same time interval. Here an irradiated sample with a dose of 10Gy was preheated to a defined temperature during 2 min, thereafter cooled back to room temperature and OSL readout was carried out. In order to eliminate the signal decreasing due to the previous readouts, the OSL signal intensity was normalized on the optical bleaching curve (see inset in Fig. 6). This final OSL signal can be ascribed to a gradual release of the captured charges under stimulation with a green light from LED source and further recombination at the Mn^2+^ related luminescent centers.

As is seen from Fig. 6, the thermal depletion process of TR-OSL intensity begins right at the room temperature showing a steep fall of the curve down to about 80 °C. This steep decrease of the TR-OSL signal is due to a strong afterglow that as described above is related to holes release from shallow traps of zinc vacancies. However, earlier studies of the thermal stability of MgGa_2_O_4_: Mn^2+^ showed no steep decrease in the low-temperature region^12^ and the curve exhibited a gradual decrease of the OSL signal. The remarkable shoulder has been observed at about 85 °C. It should be noted that the position of this shoulder corresponds to a peak position of the TSL curve of ZnGa_2_O_4_: Mn^2+^ recorded in an hour after X-ray irradiation (Fig. 3). The TR-OSL signal above 100 °C shows a plateau-like character of a curve with a small slope up to 180 °C. We can assume that some of the traps related to TL glow peaks described above are optically active and involved in the TR-OSL emission.

Optical bleaching of the TR-OSL signal versus cumulative time of stimulation is shown as an inset in Fig. 6. The cumulative stimulation time here was calculated as the multiplication of the pulse duration (100 ms) for the number of readings. As it is seen from the figure, the TR-OSL signal of ZnGa_2_O_4_: Mn^2+^ decreases after each repeated reading. In particular, after the stimulation time of 800 s, an amplitude of the integrated TR-OSL signal is decreased to about 6% of the initial signal. After the stimulation time of about 3000 s, the integrated TR-OSL signal is equal to the instrumental background corresponding the PL signal. It should be noted that the bleaching decay curve of the TR-OSL signal intensity as a function of the cumulative stimulation time in the range 0–800 s, which imitates the continuous wave OSL (CW-OSL) decay, is not a single exponential decay and can be described by a sum of two exponential components as shown in the inset in Fig. 6. However, it is also difficult to describe the bleaching curve by double exponent on the entire time interval of 0–3000 s. In this case, the curve for a time larger than 30 s is well described by generalized hyperbola function. This complex form of the TR-OSL bleaching curve indicates existence of more than one type of recombination centers involved.

## Conclusions

Zinc gallate spinel activated with 0.05 mol.% of MnO was successfully prepared by a common solid-state reaction method at 1200 °C in air. The green color emission of Mn^2+^ ions peaked at 505 nm in ZnGa_2_O_4_ at room temperature can be efficiently excited by X-rays due to the “band-to-band” transitions or UV light falling into the O^2−^ → Mn^2+^ charge-transfer band at 285 nm.

Upon X-ray exposure, ZnGa_2_O_4_: Mn^2+^ ceramics demonstrates a relatively long green afterglow that is visible to the naked eye in the dark for one hour. This afterglow is caused by few types of shallow traps that reveal itself in the thermal glow with maxima of TSL peaks at slightly above room temperature, which are dependent on the time that passed after the X-ray exposure. These shallow traps are probably related to vacancies of zinc, which are responsible for prolonged afterglow.

Analysis of the afterglow decay kinetics revealed that after the X-ray exposure switches off, the emission intensity drops to the initial afterglow level which is approximately 44% of the steady-state intensity. After 60 minutes of observation, about 3% of the steady-state intensity is still registered. After that, a half of the stored energy releases during the first day of storage that was confirmed by measurements of the residual TSL signal. It was revealed that practically all the TSL signal of the material exposed to X-rays fades during two weeks of dark storage.

Nevertheless, after the X-ray exposure a small amount of charges are captured in deep traps that are responsible for the TSL peak at about 180 °C. This particular TSL peak survives even after dark storage during a year of the irradiated samples. This observation indicates a principle opportunity to use ZnGa_2_O_4_: Mn^2+^ phosphor as a dosimetric material. However, additional studies aimed to estimate a relative sensitivity and fading characteristics of the TSL peak at 180 °C are required to be done.

It was confirmed that optical stimulation by visible light leads to much faster bleaching of the samples exposed to ionizing radiation. In such a way the optical stimulation technique can be used as an alternative to the thermal stimulation to free up the energy stored in the studied material after ionizing irradiation. The time-resolved pulsed OSL technique tested previously for other Mn^2+^-doped storage phosphors like YAlO_3_: Mn perovskite or MgGa_2_O_4_: Mn spinel, was approved here to be applicable also for OSL readout of the ZnGa_2_O_4_: Mn^2+^ spinel. In particular, a typical OSL decay lifetime for ZnGa_2_O_4_: Mn^2+^ was revealed to be from 97 to 132 ms depending on the temperature of the preheating used. This OSL lifetime can be easily resolved from the photoluminescence lifetime of Mn^2+^ ions in ZnGa_2_O_4_ being 5.1 ms.

## References

[1] Kinoshita C, Fukumoto K, Fukuda K, Garner FA, Hollenberg GW (1995). Why is magnesia spinel a radiation-resistant material?. J. Nucl. Mater..

[2] Lushchik A (2018). Creation and thermal annealing of structural defects in neutron-irradiated MgAl_2_O_4_ single crystals. Nucl. Instr. Methods Phys. Res. B.

[3] Jouini A, Yoshikawa A, Brenier A, Fukuda T, Boulon G (2007). Optical properties of transition metal ion-doped MgAl_2_O_4_ spinel for laser application. phys. stat. sol. (c).

[4] Dutta DP, Ghildiyal R, Tyagi AK (2009). Luminescent Properties of Doped Zinc Aluminate and Zinc Gallate White Light Emitting Nanophosphors Prepared via Sonochemical Method. J. Phys. Chem. C.

[5] Luchechko A, Kravets O (2017). Novel visible phosphors based on MgGa_2_O_4_-ZnGa_2_O_4_ solid solutions with spinel structure co-doped with Mn2 + and Eu3 + ions. Journal of Luminescence..

[6] Sharma SK (2014). Persistent luminescence of AB_2_O_4_: Cr^3+^ (A = Zn, Mg, B = Ga, Al) spinels: New biomarkers for *in vivo* imaging. Opt. Mater..

[7] Qin X (2016). Hybrid coordination-network-engineering for bridging cascaded channels to activate long persistent phosphorescence in the second biological window. Scientific reports..

[8] Nie J (2017). Tunable long persistent luminescence in the second near-infrared window via crystal field control. Scientific reports..

[9] Yoshimura EM, Yukihara EG (2006). Optically stimulated luminescence of magnesium aluminate (MgAl_2_O_4_) spinel. Radiat. Meas..

[10] Raj SS (2016). TL and OSL studies of carbon doped magnesium aluminate (MgAl_2_O_4_: C). Radiat. Phys. Chem..

[11] Yoshimura EM, Yukihara EG (2006). Optically stimulated luminescence: Searching for new dosimetric materials. Nuclear Instruments and Methods in Physics Research, B..

[12] Luchechko A (2018). TL and OSL properties of Mn^2+^-doped MgGa_2_O_4_ phosphor. Opt. Mater..

[13] Lengar I (2016). Radiation damage and nuclear heating studies in selected functional materials during the JET DT campaign. Fusion Engineering and Design.

[14] Ibarra A, Bravo D, Lopez FJ, Garner FA (2005). High-dose neutron irradiation of MgAl_2_O_4_ spinel: effects of post-irradiation thermal annealing on EPR and optical absorption. Journal of nuclear materials..

[15] Valiev D (2018). Luminescent properties of MgAl_2_O_4_ ceramics doped with rare earth ions fabricated by spark plasma sintering. Ceramic International..

[16] Platonenko, A., Gryaznov, D., Zhukovskii, Y. F. & Kotomin, E. A., First Principles Simulations on Migration Paths of Oxygen Interstitials in MgAl_2_O_4_. *Physica status solidi (b)*. 1800282 (2018).

[17] Kirm M. *et al*. Luminescent materials with photon multiplication. Optical Inorganic Dielectric Materials and Devices (eds Krumins, A., Millers, D. K., Sternberg, A. & Spigulis, J.) *Proc. SPIE*. **2967**, 18–23 (1997).

[18] Gritsyna VT, Kazarinov YG, Kobyakov VA, Sickafus KE (2002). Defects and radiation induced electronic processes in magnesium aluminate spinel of different compositions. Rad. Eff. Def. Solids..

[19] Wang SF (2015). A comparative study of ZnAl_2_O_4_ nanoparticles synthesized from different aluminum salts for use as fluorescence materials. Scientific reports..

[20] Zhou Q (2018). Mn^2+^ and Mn^4+^ red phosphors: synthesis, luminescence and applications in WLEDs. A review. Journal of Materials Chemistry C..

[21] Antuzevics A, Rogulis U, Fedotovs A, Popov AI (2018). Crystalline phase detection in glass ceramics by EPR spectroscopy. Low Temperature Physics..

[22] Poort SHM, Cetin D, Meijerink A, Blasse G (1997). The Luminescence of Mn^2+^ Activated ZnGa_2_O_4_. J. Electrochem. Soc..

[23] Uheda K, Maruyama T, Takizawa H, Endo T (1997). Synthesis and long-period phosphorescence of ZnGa_2_O_4_: Mn^2+^ Spinel. Journal of Alloys and Compounds..

[24] Yu CF, Lin P (1996). Manganese-activated luminescence in ZnGa_2_O_4_. J. Appl. Phys..

[25] Kim JS, Kim JS, Kim TW, Kim SM, Park HL (2005). Correlation between the crystalline environment and optical property of Mn^2+^ ions in ZnGa_2_O_4_: Mn^2+^ phosphor. Applied Physics Letters.

[26] Bøtter-Jensen, L., McKeever, S. W. & Wintle, A. G. Optically Stimulated Luminescence Dosimetry. *Elsevier*. pp. 1–355 (2003).

[27] Yukihara, E. G. & McKeever, S. W. S. Optically Stimulated Luminescence: Fundamentals and Applications. John Wiley & Sons. pp. 1–362 (2011).

[28] Yukihara EG, McKeever SW, Akselrod MS (2014). State of art: Optically stimulated luminescence dosimetry – Frontiers of future research. Radiat. Meas..

[29] Zhydachevskii Y, Suchocki A, Berkowski M, Zakharko Y (2007). Optically stimulated luminescence of YAlO_3_: Mn^2+^ for radiation dosimetry. Radiat. Meas..

[30] Zhydachevskii Y (2016). Time-resolved OSL studies of YAlO_3_: Mn^2+^ crystals. Radiat. Meas..

[31] Dotzler CJ, Williams GVM, Edgar A (2007). Thermoluminescence, photoluminescence and optically stimulated luminescence properties of X-ray irradiated RbMgF_3_: Mn^2+^. phys. stat. sol. (c).

[32] Williams GVM, Schuyt JJ, Madathiparambil AS (2018). The effect of Mn concentration on the luminescence properties of NaMgF_3_: Mn: defect/Mn complex photoluminescence, radioluminescence, and optically stimulated luminescence for radiation dose monitoring. Opt. Mater..

[33] Bakshi AK (2009). Study on TL and OSL characteristics of indigenously developed CaF_2_: Mn phosphor. Nucl. Instr. Methods Phys. Res. B.

[34] Danilkin M (2010). Storage mechanism and OSL-readout possibility of Li_2_B_4_O_7_: Mn (TLD-800). Radiat. Meas..

[35] Yanagida T, Fukuda K, Okada G, Watanabe K, Kawaguchi N (2017). Ionizing radiation induced luminescence properties of Mn-doped LiCa(Al,Ga)F_6_. J. Mater. Sci..

[36] Zhydachevskyy, Y. *et al*. Thermally induced fading of Mn-doped YAP nanoceramics. *Journal of Physics**:**Conference Series*, **987**, No. 1, 012009, IOP Publishing (2018).

[37] Piskunov S, Isakoviča I, Popov AI (2018). Electronic structure of Mn_Al_^3+^-and Mn_Al_^2+^-doped YAlO_3_: Prediction from the first principles. Optical Materials..

[38] Katsumata T, Takeuchi H, Komuro S, Aizawa H (2018). X-ray detector based on Mn doped MgAl_2_O_4_ and Si photodiode. Review of Scientific Instruments..

[39] Dotzler C (2006). The effect of x-ray, γ-ray, and UV radiations on the optical properties of RbCdF_3_: Mn^2+^. Journal of applied physics..

[40] Luchechko A, Kravets O, Syvorotka II (2017). Optical and luminescence spectroscopy of zinc gallate phosphors co-doped with manganese and europium ions. Spectroscopy Letters..

[41] Bulur E, Saraç BE (2013). Time-resolved OSL studies on BeO ceramics. Radiat. Meas..

[42] Luchechko A, Kravets O, Kostyk L, Tsvetkova O (2016). Luminescence spectroscopy of Eu^3+^ and Mn^2+^ ions in MgGa_2_O_4_ spinel. Radiat. Meas..

[43] Yi SS (2002). Luminescence characteristics of ZnGa_2_O_4_ thin film phosphors grown by pulsed laser deposition. Mater. Lett..

[44] Kravets, O. *et al*. Structure, morphology and optical-luminescence investigations of spinel ZnGa_2_O_4_ ceramics co-doped with Mn^2+^ and Eu^3+^ ions. *Applied Nanoscience*. 1–9 (Article in Press, Accepted Manuscript) (2018).

[45] Sharma SK (2014). Interplay between chromium content and lattice disorder on persistent luminescence of ZnGa_2_O_4_: Cr^3+^ for *in vivo* imaging. J. Lumin..

[46] Matsui H, Xu CN, Akiyama M, Watanabe T (2000). Strong mechanoluminescence from UV-irradiated spinels of ZnGa_2_O_4_: Mn and MgGa_2_O_4_: Mn. Japanese Journal of Applied Physics..

